# Institutional Notes and News

**Published:** 1910-08-13

**Authors:** 


### Institutional Notes and News.

**Shadwell Children's Hospital.**

Lord Erroll presided last week at the half-yearly court of governors of the East London Hospital for Children, Glamis Road, Shadwell, E. The report again drew attention to the financial position. No windfalls had come in in the shape of large legacies, with the result that the management is faced with a considerable debt, but there are hopes that this debt will be discharged in full. The receipts for the six months for the hospital amounted to £3,516, against £4,587 in the corresponding half-year of 1909, while the expenditure exceeded the income by £1,900. The total number of in-patients during the half-year was 984 and the out-patients 17,146, with 45,713 attendances. At the convalescent home 143 patients were treated. It was stated that the plans for the improvements in the out-patient department had been approved by the King's Fund. The plans comprised an operating theatre, with preparation, recovery, and sterilising rooms annexed, two new rooms for the casualty officers, and an office for the almoner. At the end of the June half-year the governors had to borrow £3,000 from the bankers, but since then a legacy amounting to £5,600 had been received. The hospital is doing excellent service in the East End, and is one of the most deserving of children's hospitals in the Metropolis.

**The New Edmonton Infirmary.**

The official opening of the Edmonton Infirmary took place last month, by Sir William Collins, M.P. The Chairman and Vice-Chairman of the Board and the Chairman and Vice-Chairman of the Infirmary Committee received Sir William and Lady Collins at the entrance to the infirmary. Sir W. J. Collins then unlocked the principal door of the infirmary and proceeded, with the Guardians and the company assembled, which numbered 300, to one of the large wards, where Mr. Metivier, Chairman of the Infirmary Committee, spoke. He paid a compliment to the architect, the late Mr. Stuart Hill, who did not live to see his work finished, and said the infirmary was now quite separate from the workhouse, and that patients could now go there without feeling pauperised. Sir William Collins then declared the building open, adding that the Guardians were to be congratulated on the cost of the establishment. The contract was £72,724, and the cost of the building was considerably under that amount. Soon, he hoped, Poor-Law infirmaries would be called municipal hospitals, and Guardians, he thought, were dying hard. He believed there were now some 6,500 professional nurses employed under the Poor Law, and in 1820 there were only about 200. Mr. Cole, present Chairman of the Board, in proposing a vote of thanks to Sir William and Lady Collins, said he thought that Boards of Guardians would never be abolished. He said it was the cheapest infirmary of its size ever built, and that Edmonton was one of the most economical Boards in the country. The Rev. W. Haines seconded the vote of thanks, and hoped Edmonton Infirmary would now rank with the hospitals. He could not understand why such distinctions were made between infirmaries and hospitals; both were supported by the public, the only difference being that one was voluntary support and the other a levied rate. The Rev. David Fotheringham reminded the meeting that during the ten years when he was Chairman this new infirmary was talked of and planned in conjunction with the late master and matron, who had done so much for the comfort and welfare of the inmates, and he for one could not go away without a word of praise for them. The infirmary is to accommodate 384 patients; there are twelve wards, or six double wards, two of each name, and each sister is to have charge of two of these, each containing twenty-eight beds; also four observation wards, with two beds apiece. There is a bathroom containing two baths at the end of each ward, also a ward kitchen and a general store. The lavatories are in front the centre of the wards, and a day room is partitioned off from the ward, where convalescent patients can read and dine away from the general. To carry the food to the wards are waggons fitted with hot-water tanks. There are receiving wards also for each sex, and a suite of rooms for the reception, examination, and cleansing of every patient before admission.

**Swansea Hospital.**

The ninety-third annual meeting of Swansea Hospital was largely concerned with the consideration of an appeal for £25,000 in order to increase the number of beds. Owing to difficulties about the additional land required, the board has fallen back upon the expedient of raising the existing buildings. During the last ten years the number of in-patients has increased considerably and the number of out-patients has doubled. One of the hopeful facts of the financial situation is the heroic way in which the working classes have increased their subscriptions; but unfortunately, though probably not deliberately, the richer classes have at the same time reduced theirs, which is fair neither on the working men nor on the hospital. Mr. T. W. Hughes was complimented on his work as secretary, and this work is not likely to be diminished till this appeal is off the hospital's hands. It is hoped that the generous example set by Sir John Llewelyn and Miss Talbot in giving £900 and £800 respectively to renovate the wards named after them will be followed by the classes which they represent. Our account of the annual meeting has been held over unavoidably, because those superintendents who send us up-to-date accounts for this section of The Hospital necessarily have the first claim on our space.

